# Synthesis Method for Thiosulfonate and Report of Its Insecticidal Activity in *Anagasta kuehniella* (Lepidoptera: Pyralidae)

**DOI:** 10.3390/ijms131115241

**Published:** 2012-11-19

**Authors:** Edson dos A. dos Santos, Fernando Gonçalves, Paulo César Prado, Daniele Y. Sasaki, Dênis P. de Lima, Maria Lígia Rodrigues Macedo

**Affiliations:** 1 Laboratory of Synthesis and Transformation of Organic Molecules, Chemistry Section of CCET, Federal University of Mato Grosso do Sul, CP 549, CEP 79070-900, Campo Grande-MS, Brazil; E-Mails: edsonanjos@hotmail.com (E.A.S.); dpireslima@gmail.com (P.C.P.); dpires@gmail.com (D.P.L.); 2 Laboratory of Protein Purification and its Biological Functions, CCBS, Federal University of Mato Grosso do Sul, CP 549, CEP 79070-900, Campo Grande-MS, Brazil; E-Mails: fhmgoncalves@yahoo.com.br (F.H.M.G.); danieleys@hotmail.com (D.Y.S.)

**Keywords:** insect, pests insecticidal activity, growth retardation, thiosulfonates

## Abstract

Insect pests have caused economic losses valued at billions of dollars in agricultural production. *Anagasta kuehniella* (Zeller), the Mediterranean flour moth, is of major economic importance as a flour and grain feeder and is often a severe pest in flourmills. This study provides a suitable route for the direct preparation of thiosulfonates **2** and **3** from thiols, under mild conditions, with good yields; these thiosulfonates were tested for their regulatory effect on insect growth. The chronic ingestion of thiosulfonates resulted in a significant reduction in larval survival and weight. In addition, the tryptic activity of larvae was sensitive to these thiosulfonates. Results suggest that thiosulfonates **2** and **3** have a potential antimetabolic effect when ingested by *A. kuehniella*. The use of AgNO_3_/BF_3_·OEt_2_ and Al(H_2_PO_4_)_3_/HNO_3_ provides a suitable route for the direct preparation of thiosulfonates from thiols under mild conditions with good yields. These thiosulfonates were toxic for *A. kuehniella* larvae, suggesting their potential as biotechnological tools.

## 1. Introduction

Insect pests have caused economic losses valued at billions of dollars in agricultural production [1], achieving values of higher than 10% of the total production of the country [2]. Among these insects, *Anagasta kuehniella* (Zeller), the Mediterranean flour moth, is found worldwide and is of major economic importance as a flour and grain feeder; this species is often a severe pest in flourmills [3] and, particularly in stored grains such as fruits and nuts. Few stored and dried vegetable products are safe from this small and voracious moth: nuts, fruits, chocolates, biscuits, cakes, jellies, and candies are also consumed by it [4]. Controlling these insects generally requires the use of chemical insecticides, such as malathion, pirimiphos-methyl, chlorpyrifos-methyl, pyrethrum, deltamethrin, methoprene and the fumigant, phosphine, which are all toxic to humans and domestic animals and harmful to the environment [5]. However, consumer concern is increasing regarding insecticide residues in processed cereal products, the occurrence of insecticide-resistant insect strains and the precautions necessary for this work [6].

The arylthiosulfonates **1**, **2** and **3** have shown valuable biological activities, such as cytotoxic [7] and fungicide [8,9] abilities. This class of thiosulfonates displays insecticidal activity in azuki bean weevils and rice stem borer larvae [10,11], but the insecticidal activity of these thiosulfonates for *A. kuehniella* larvae is not yet known.

The present study deals with two new methods for the synthesis of thiosulfonates and examines the effect of thiosulfonates **2** and **3** on the development and survival of *A. kuehniella* larvae. We also examined the effects of the thiosulfonates on nutritional indices and on soluble trypsin endoproteases, the major enzymes involved in protein digestion.

## 2. Results and Discussion

### 2.1. Experimental Methods of Chemistry

Reactions for the preparation of thiosulfonates generally require the coupling of thiols with arylsulfonic [12], arylsulfinic acids [13] or sulfonyl chlorides [14]. Thiosulfonates can also be prepared by oxidation of disulfides [15]. However, few reactions have reported their preparation from thiols in good yields [16,17]; for such reactions, the production of dissulfides and sulfonodithioic *O*-acid can also occur [18].

We, herein, report a new application of silver nitrate (AgNO_3_) with boron trifluoride etherate (BF_3_·OEt_2_) and aluminium dihydrogen phosphate (Al(H_2_PO_4_)_3_) as selective and efficient reagents for the oxidative coupling of thiophenol (**1**), 4-methylthiophenol (**2**) and 4-methoxythiophenol (**3**) to corresponding thiosulfonates (Table 1).

#### 2.1.1. General Procedure with AgNO_3_/BF_3_·OEt_2_ and Al(H_2_PO_4_)_3_/HNO_3_

**Figure 1:**
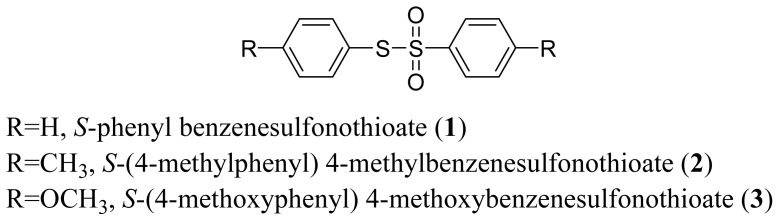
Structure of arylthiosulfonates **1**, **2** and **3**.

These methods are known to be nitration agents for phenolic compounds [19,20]. The reaction of AgNO_3_ with BF_3_·OEt_2_ in acetonitrile and in a nitrogen atmosphere at room temperature resulted in yields of 72%–75%. Employing the reaction with Al(H_2_PO_4_)_3_, the solid acid [21] with concentrated nitric acid at room temperature resulted in yields of 56%–61%. The use of this reagent provides the possibility of the catalytic oxidative coupling of thiols under mild conditions. The disulfides produced are isolated with excellent purity and good yields (Figure 1).

### 2.2. Effects of Thiosulfonates **2** and **3** on the Development of *A. kuehniella*

**Figure 2:**
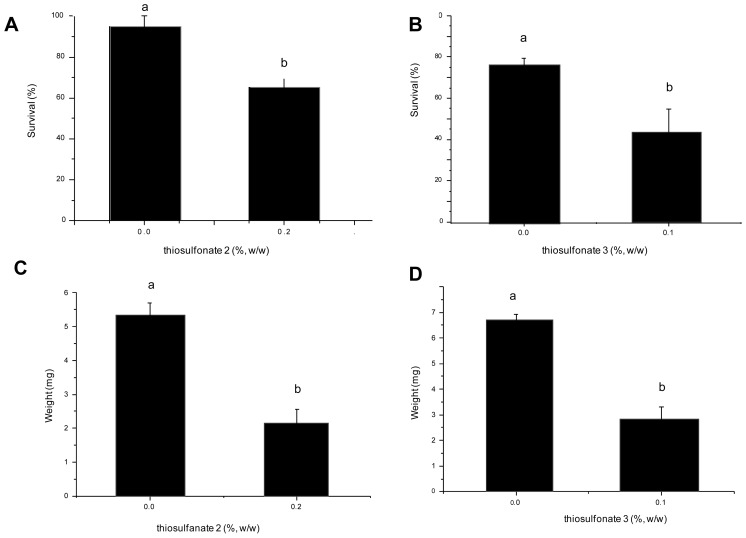
Effect of dietary thiosulfates (**2** and **3**) on the weight and survival of *A. kuehniella* larvae. Different letters denote a significant difference between the treatments (ANOVA, *p* < 0.05).

The effect of the thiosulfonates **2** and **3** on the larval development of *A. kuehniella* was monitored by feeding the larvae on an artificial diet and then determining the number and mass of surviving fourth instar larvae. Figure 2 shows the effect of thiosulfonates **2** and **3** on the survival and weight of *A. kuehniella* larvae at the fourth instar. The survival of *A. kuehniella* larvae feeding on diets with thiosulfonates **2** and **3** were approximately 32% and 42%, when compared to survival on the control diet (Figure 2a,b), respectively; whereas diets containing thiosulfonates **2** and **3** reduced the weight of the larvae by 62% and 57% when compared to the weight of larvae on the control diet (Figure 2a,b). Concentrations of above 0.2% and 0.1% thiosulfonates **2** and **3**, respectively, caused 100% *A. kuehniella* mortality.

#### 2.2.1. Nutritional Parameters

Nutritional analyses revealed that thiosulfonates **2** and **3** presented a toxic effect when ingested by larvae. The thiosulfonates **2** and **3**, when incorporated in an artificial diet at 0.2% and 0.1%, respectively, reduced ECI and ECD and increased AD and metabolic cost (CM) for *A. kuehniella* larvae, when compared with the control. Thiosulfonate **2** significantly decreased both ECI and ECD by 21% and 45%, respectively, and AD and CM were increased by 6.5% and 5.5%, respectively, when compared with larvae of *A. kuehniella* that were reared on control diets. Thiosulfonate **3** decreased both ECI and ECD by 45% and 52%, respectively, and AD and CM were increased by 14% and 7%, respectively, when compared with larvae of *A. kuehniella* that were reared on control diets.

The AD value for larvae of *A. kuehniella*, in the present study, was increased throughout the feeding period of the experiment; this finding suggests that, during this treatment, the food remained for a greater time in the insect’s gut to allow the detoxification of the thiosulfonates. A greater AD would help to meet the increased demand for nutrients [22,23] and compensate for the deficiency in food stuff conversion (reduction in ECI and ECD), perhaps by diverting energy from biomass production to detoxification [20]. This behavior has also been observed by others [4,24]. A drop in ECI indicates that more food is being metabolized for energy and less is being converted to body mass, *i.e.*, growth of the insect [14]. ECD also decreases as the proportion of digested food metabolized for energy increases [25]. We suggest that the reduction in ECD is likely to result from a reduction in the efficiency to convert foodstuffs into growth, perhaps by a diversion of energy from the production of biomass into detoxification of thiosulfonates increase in costs. Results show that there were problems in the conversion of the food assimilated, suggesting that during this treatment, the diet remained in the gut of the insect for a longer time to allow detoxifying of the effects caused by the thiosulfonates **2** and **3**[3]. There was probably a greater expenditure of energy from the diet to be used in the process of degradation of thiosulfonates present and also the maintenance of the vital processes of the insect.

#### 2.2.2. Tryptic Activity

**Figure 3:**
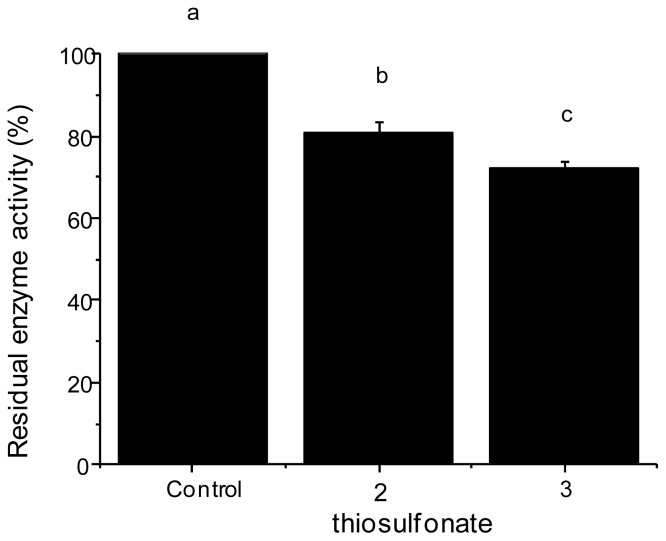
Inhibition by thiosulfonates **2** and **3** of the midgut proteolytic activity (assayed with BAPNA) of fourth instar *A. kuehniella* larvae. Different letters indicate a significant difference between the treatments (ANOVA, *p* < 0.05).

Studies were undertaken to evaluate the action of these thiosulfonates on the tryptic activity of the larvae of *A. kuehniella*, utilizing BAPNA as a synthetic substrate. Figure 3 shows the effects of different doses of thiosulfonates **2** and **3** (1 μg) on the in vitro activity of digestive proteinases from fourth instar larvae. The thiosulfonates **2** and **3** altered the tryptic activity of these proteinases by 20% and 28%, respectively.

**Figure 4:**
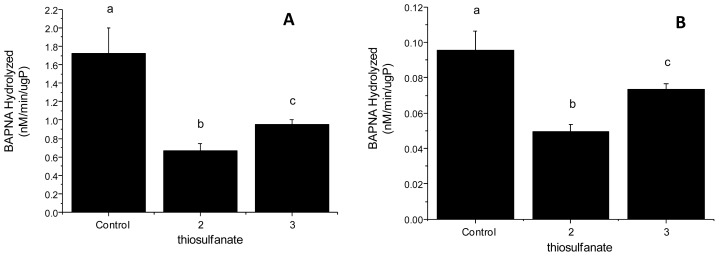
Trypsin-like activities in fourth instar larvae fed on a control artificial diet and containing thiosulfonates **2** (0.2%) and **3** (0.1%). (**A**) Enzymatic activity of the midgut; (**B**) Enzymatic activity of the feces. Trypsin activity was evaluated using BAPNA as substrate. Different letters denote a significant difference between the treatments (ANOVA, *p* < 0.05).

Larvae reared on artificial diets containing thiosulfonates **2** (0.2%) and **3** (0.1%) were observed for trypsin proteinase activity. The enzyme assay showed that larvae fed on thiosulfonates **2** and **3** resulted in low levels of trypsin activity in the gut and in fecal material (Figure 4a,b). Tryptic activities in the midgets of fourth instar *A. kuehniella* larvae reared on artificial diets containing thiosulfonates **2** and **3** were altered by 59% and 41%, respectively (Figure 4a), and the tryptic activities in the feces were altered by 47% and 26%, respectively (Figure 4b), when compared with those of larvae on control diets. The decrease in the trypsin activities of feces of thiosulfonate-fed larvae suggest that thiosulfonates did not cause the rupture of the peritrophic membrane of *A. kuehniella*.

These thiosulfonate compounds presented a toxic effect on *A. kuehniella* larvae as they can block the trypsin enzyme involved in this insect’s digestion. In addition, all trypsin proteinase activities in thiosulfonate-fed larvae were sensitive to thiosulfonates, indicating that no novel proteolytic form resistant to thiosulfonates was induced in larvae reared on a diet containing these compounds. Similar results were observed with a Kunitz-type inhibitor (APTI) of 21 KDa isolated from the seeds of *Adenanthera pavonina*, the seed proteinase inhibitors act as a part of the plant defensive system against pest via inhibition of their proteolytic enzymes [3,26].

The TLCK (tosyl-l-lysine chloromethyl ketone) is an irreversible inhibitor of trypsin and has a sulfonyl group in its molecular structure. This compound binds covalently to a serine at the active site of trypsin, inhibiting its enzymatic action; the sulfonyl group does not participate directly in this connection [27]. Regarding the tested arylthiosulfonates, we suggest that they interact with trypsin but not by a covalent bond as it was observed that only a decreasing of trypitic activity, which might be due to hydrogen bonding to hydrophilic amino acids in the active site of this enzyme [27]. This would explain the greater toxicity of thiosulfonate **3** compared to thiosulfonate **2**, since the latter compound has methoxyl groups.

## 3. Experimental Section

### 3.1. Experimental Methods of Chemistry

All melting points were determined using the Uniscience of Brazil Mod. 498 equipment. NMR spectra were recorded in CDCl_3_ solutions on a Bruker DPX-300 instrument. All chemical shifts (δ) were referenced to TMS. The mass spectra were measured using a Shimadzu GCMS-QP2010 Plus gas chromatograph mass spectrometer. The reactions were monitored by TLC on silica gel-precoated aluminum sheets (Alugram^®^ Sil G/UV_254_, Macherey-Nagel). Solvents employed in the reactions and silica gel column chromatography were previously purified and dried according to procedures found in the literature [28]. Purification of compounds was performed by column chromatography, using stationary phase silica gel 60 (0.035–0.075 mm) from Acros Organics. All reagents were of analytical grade.

#### 3.1.1. General Procedure with AgNO_3_/BF_3_·OEt_2_

Thiol (7 mmol) was dissolved in anhydrous acetonitrile (23 mL) in a round-bottom flask under a nitrogen atmosphere, before adding AgNO_3_ (7.7 mmol) and BF_3_·OEt_2_ (0.8 mL) slowly to the solution. The reaction mixture was stirred for 17 h at room temperature. The reaction solution was then quenched with ice water, and the pH was increased to 7 by the careful addition of sodium bicarbonate saturation solution. The resulting solution was filtered and the filtrate was extracted with ethyl acetate, the organic layer was washed with distilled water and brine. The organic layer was dried over magnesium sulfate and the solvent was evaporated at reduced pressure; the resulting material was purified by flash chromatography using hexane-ethyl acetate (8:2) as eluent.

#### 3.1.2. General Procedure with Al(H_2_ PO_4_)_3_/HNO_3_

The Al(H_2_PO_4_)_3_ catalyst was elaborated by addition of 85% H_3_PO_4_ (16 mL) in neutral Al_2_O_3_ (39 mmol) in a porcelain crucible. The mixture was heated at 220 °C in a muffle for 2 h. The mixture was then placed in a vacuum desiccator and cooled to room temperature. The catalyst, thus prepared, was then stored in sealed sample vial.

To thiol (32 mmol) was added Al(H_2_PO_4_)_3_ (0.16 mmol) and concentrated HNO_3_ (3 mL). The reaction mixture was stirred for 24 h at room temperature. The mixture was transferred to a dropping funnel, and ethyl acetate was added. The pH was increased to 7 by the careful addition of sodium bicarbonate saturation solution. The organic layer was washed with distilled water, and brine, and dried over magnesium sulfate. The solvent was evaporated at reduced pressure, and the resulting material was purified by flash chromatography using hexane-ethyl acetate (8:2) as eluent.

#### 3.1.3. *S*-phenyl Benzenesulfonothioate (**1**)

White solid; mp 36–38 °C, lit. 35–37 °C [29]. ^1^H NMR (300 MHz, CDCl_3_) δ 7.34 (m, 4H), 7.45 (m, 3H), 7.56 (m, 3H).^13^C NMR (CDCl_3_) δ 127.5 (CH), 127.8 (C), 128.7 (CH), 129.4 (CH), 131.3 (CH), 133.5 (CH), 162.1 (C), 163.5 (CH), 142.9 (C). MS *m*/*z* (%): 250 [M^+^] (32); 141 (33); 125 (100); 109 (36); 77 (76).

#### 3.1.4. *S*-(4-Methylphenyl) 4-Methylbenzenesulfonothioate (**2**)

White solid; mp 77–78 °C, lit. 77–78 °C [30]. ^1^H NMR (300 MHz, CDCl_3_) δ 2.63 (s, 3H), 2.41 (s, 3H), 7.13 (d, *J* = 8.0 Hz, 2H), 7.20 (d, *J* = 8.0 Hz, 2H), 7.23 (d, *J* = 8.2 Hz, 2H), 7.44 (d, *J* = 8.2 Hz, 2H). ^13^C NMR (CDCl_3_) δ 21.4 (CH_3_), 21.6 (CH_3_), 124.5 (C), 127.5 (CH), 129.3 (CH), 130.1 (CH), 136.4 (CH), 140.4 (C), 142.0 (C), 144.5 (C). MS *m*/*z* (%): 278 [M^+^] (30); 155 (20); 139 (100); 124 (32); 123 (34); 91 (87).

#### 3.1.5. *S*-(4-Methoxyphenyl) 4-Methoxybenzenesulfonothioate (**3**)

White solid; mp 84 °C, lit. 84–85 °C [31]. ^1^H NMR (300 MHz, CDCl_3_) δ 3.81 (s, 3H), 3.85 (s, 3H), 6.83 (d, *J* = 8.6 Hz, 2H), 6.86 (d, *J* = 8.6 Hz, 2H), 7.25 (d, *J* = 8.8 Hz, 2H), 7.48 (d, *J* = 8.8 Hz, 2H). ^13^C NMR (CDCl_3_) δ 55.4 (CH_3_), 55.6 (CH_3_), 113.7 (CH), 114.8 (CH), 118.8 (C), 129.8 (CH), 134.8 (C), 138.3 (CH), 162.1 (C), 163.4 (C). MS *m*/*z* (%): 310 [M^+^] (16); 171 (13); 155 (63); 140 (56); 139 (100); 125 (35).

### 3.2. Insect

A culture of flour moths (*A. kuehniella* (Zeller); Phycitinae, Pyralidae, Lepidoptera) was originally supplied by Dr. J.R.P. Parra (Laboratório de Biologia dos Insetos, Escola Superior de Agronomia “Luiz de Queiroz”, Universidade São Paulo, Piracicaba, SP, Brazil). The insects were housed at 28 ± 1 °C, at a relative humidity of 65%–75% (16 h photophase) and routinely maintained on a standard diet of wheat germ.

#### 3.2.1. Midgut Preparation

Fourth instar larvae were cold-immobilized and dissected in cold 250 mM NaCl. The midguts were surgically removed from the larvae using tweezers. Only actively feeding larvae with guts that were filled with food were used. The gut portion taken was posterior to the proventriculus and anterior to the malpighian tubules. After removing all extraneous tissue and freeing the lumen of its contents by rinsing in 250 mM NaCl, the midgut tissues were homogenized in cold distilled water in a hand-held Potter-Elvehjem homogenizer immersed in ice. Midgut homogenates were centrifuged at 17,000× *g* for 20 min at 4 °C and the supernatants were collected in a known volume of appropriate buffer and used immediately as enzyme sources for enzymatic assays, and when necessary were stored at −20 °C.

#### 3.2.2. Fecal Pellet Preparations

Feces of the caterpillars were collected during the experiment and frozen (−20 °C). When necessary, they were macerated, homogenized in 200 mM Tris-HCl buffer (Tris-Hydroxymethylaminomethane), pH = 8.5, centrifuged at 20,000× *g* for 30 min at 4 °C and supernatants were used for *in vitro* enzymatic assays.

#### 3.2.3. Enzyme Assays

Trypsin-like enzymes of whole midgut extracts and fecal samples from *A. kuehniella* larvae, fed on diets containing thiosulfonates (**2** and **3**) and control diets were assayed using the synthetic substrate, *N*-benzoyl-dl-arginine *p*-nitroanilide (BAPNA), as described by Erlanger [32]. The linearity of the relationship between the changes in absorbance with time was checked to ensure that substrate concentrations were not limiting. Substrate and enzyme controls were run to ensure the validity of sample absorbance readings.

### 3.3. Effects of Thiosulfonates **2** and **3** on the Development of *A. kuehniella*

To examine the effects of thiosulfonates **2** and **3** on *A. kuehniella* development, larvae up to the fourth instar were fed on an artificial diet containing thiosulfonates **2** and **3** at concentrations of 0.1%–1.0% (*w*/*w*). A control meal without thiosulfonates was also prepared. For each treatment, four neonate larvae were placed in clear plastic, airtight containers. Each treatment was repeated fifteen times (*n* = 100). Following incubation until forth instar at 28 °C and 65%–70% relative humidity, the weight and number of larvae were determined.

### 3.4. Nutritional Parameters

Nutritional parameters were compared among fourth instar larvae exposed to thiosulfonates (**2** and **3**) or a control diet. The larvae, feces, and remaining uneaten food were separated using a microscope, dried and weighed. Nutritional indices of consumption, digestion and utilization of food were calculated, as described by literature [33,34]. The nutritional indices, namely efficiency of conversion of ingested food (*ECI*), efficiency of conversion of digested food (*ECD*) and approximate digestibility (*AD*) were calculated as follows:

ECI=(ΔB/I)×100;ECD=[ΔB/(I-F)]×100;and AD=[(I-F)I]×100

where *I* = weight of food consumed, *ΔB* is change in body weight, and *F* = weight of feces produced during the feeding period. Metabolic cost (*CM*) was calculated as: 100*ECD*.

## 4. Conclusions

The use of AgNO_3_/BF_3_·OEt_2_ and Al(H_2_PO_4_)_3_ provides a suitable route for the direct preparation of thiosulfonates from thiols under mild conditions with good yields. These thiosulfonates were toxic for *A. kuehniella* larvae, suggesting their potential as biotechnological tools.

## Figures and Tables

**Table 1:** Reactions of thiols for the formation of thiosulfonates with two catalysts.

Thiols	Product	Yield (%) of procedure	

		i a	ii b
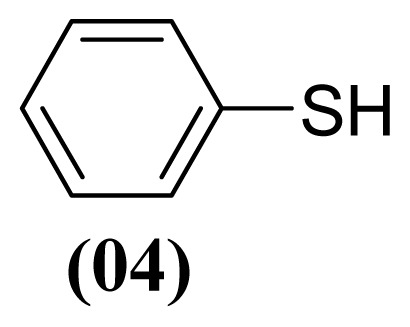	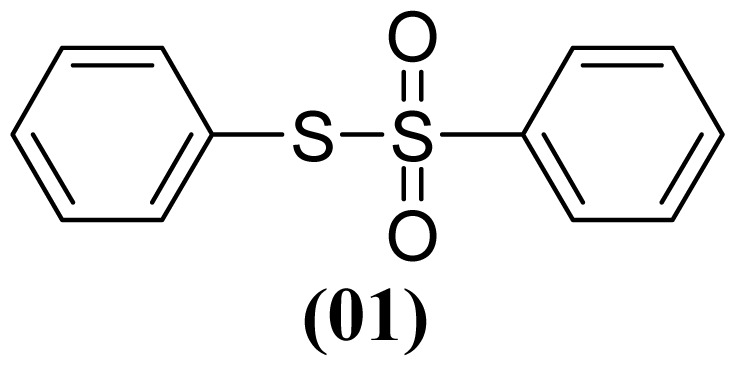	72	56
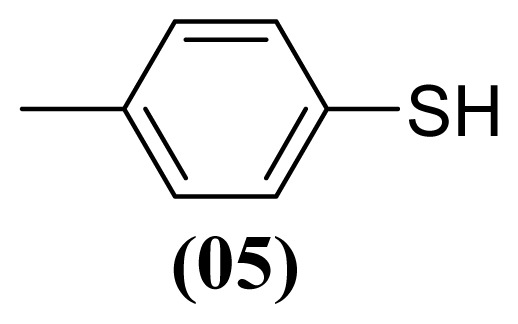	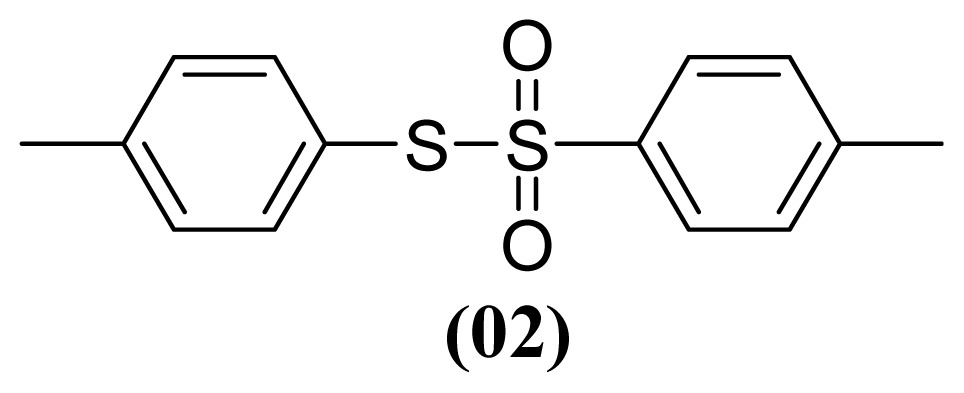	76	59
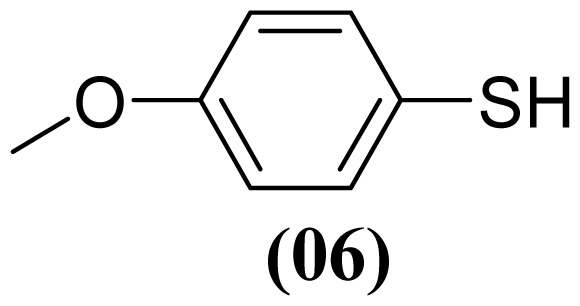	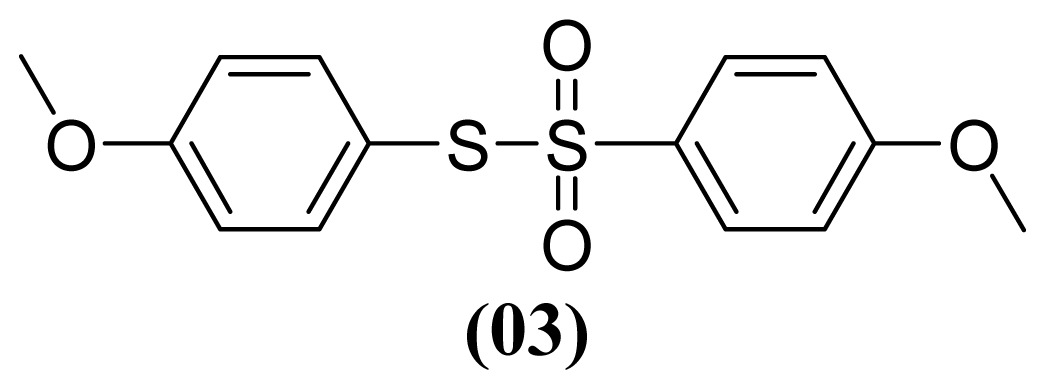	75	61

a AgNO_3_, BF_3_·OEt_2_, ACN (dry), N_2_, r.t., 17 h;

b Al(H_2_PO_4_)_3_, HNO_3_, r.t., 24 h.
